# Sequencing and Analysis of Chicken Segmented Filamentous Bacteria Genome Revealed Unique Avian-Specific Features

**DOI:** 10.3390/microorganisms14020341

**Published:** 2026-02-02

**Authors:** Jared Meinen-Jochum, Viswanathan Satheesh, Rick E. Masonbrink, Jonathan Rodriguez-Gallegos, David A. Wright, Andrew J. Severin, Melha Mellata

**Affiliations:** 1 Department of Food Science and Human Nutrition, Iowa State University, Ames, IA 50011, USA; 2 Interdepartmental Microbiology Graduate Program, Iowa State University, Ames, IA 50011, USA; 3 Office of Biotechnology, Genome Informatics Facility, Iowa State University, Ames, IA 50011, USA; 4 Office of Biotechnology, DNA Facility, Iowa State University, Ames, IA 50011, USA

**Keywords:** segmented filamentous bacteria (SFB), genome annotation, host–bacteria interaction, SFB flagella

## Abstract

Segmented filamentous bacteria (SFB) are host-specific, immune-modulating microorganisms that colonize the small intestine of various vertebrate species, playing a crucial role in stimulating immune maturation during early life. Previous research on the genomes of SFB from humans, rats, and mice has revealed significant differences among SFB strains associated with various hosts, suggesting that their evolution is closely linked to their relationships with specific hosts. However, the genome of SFB from chickens has not been extensively investigated. In this study, we present the metagenomic reconstruction of an SFB genome derived from the ileum of layer Lohmann Select Leghorn (LSL) chickens. We utilized Hi-C sequencing techniques to assemble the LSL-SFB and annotate the avian SFB from both turkeys and chickens. Our reference-guided consensus assembly, followed by Hi-C scaffolding, produced a high-quality genome for LSL-SFB. Our pangenomic analysis revealed substantial conservation of core gene clusters among mammalian SFB strains, but we also identified a distinct repertoire of genes in chicken and turkey SFB. Furthermore, metabolic network analysis indicated a reduced capacity for biosynthesis, signifying an increased reliance on the host, as shown by the absence of key biosynthetic and utilization pathways. We also discovered a unique flagellin subunit (*fliC-2*) in chicken SFB from different genetic lines and confirmed its interaction with the chicken flagellin receptor, Toll-like receptor five. This study provides the first high-quality genome and annotation of LSL-SFB, alongside that of turkeys, offering valuable insights into the mechanisms of host specificity and adaptation. Understanding the interactions between host-specific SFB and their hosts, as well as their role in promoting immune maturation, is essential for improving intestinal health.

## 1. Introduction

The gut mucosa is home to transient bacteria and bacteria that are intimately associated with the host. Of these, *Candidatus arthromitus* (segmented filamentous bacteria; SFB) are key drivers of the early maturation of the intestinal immune system, as demonstrated in mice [1,2,3] and by our team in chickens [4,5,6]. SFB are anaerobic spore-forming bacteria found in the gut of humans, mice, rats, chickens, turkeys, and many other vertebrate species [7,8,9] but are host specific [10]. Based on phylogenetic analyses, SFB are closely related to *Clostridia* species [11].

In early life, SFB colonize the distal small intestine in vertebrates and intimately bind to the epithelial tissue without causing host pathology [12,13]. Instead, the close association induces an IgA response, stimulates T cell differentiation, and upregulates intestinal innate defense mechanisms [14,15,16]. Recently, the availability of the full (human and mouse) and draft (turkey) genomes has shown the distinct relation between SFB isolated from humans, murine hosts, and turkeys [17,18]. However, the genome of SFB isolated from chickens and its relation to SFB obtained from other vertebrate species remains unstudied.

In chicken production, there are two distinct genetic lines separated by their purpose for either meat production (broilers) or egg production (layers) [19]. Within these genetic lines, broilers and layers can be separated by desirable phenotypic differences for certain production environments [20]. SFB have been detected in both layers and broilers, but these SFB must come from the environment at an early age, as eggs are separated and sterilized in rearing facilities. Our lab has recently demonstrated that SFB sourced from layer chickens are able to colonize the ileum of their genetically diverse relative broiler chickens [6]. This colonization resulted in comparable immunological maturation in the gut of broilers to specific pathogen-free (SPF) layer chickens [5,6,12].

The study of SFB *in vitro* has been greatly hindered by its limited ability to grow outside of mono-colonized mice [1,21]. Through advancements in metabolomic and culturomic techniques, studies of unculturable bacteria like SFB, their cross-talk with the host through the production and uptake of certain metabolites, and their role in their microbial community have made large strides [22,23,24]. However, little is known about SFB’s metabolic requirements and the differences between SFB isolated from different hosts. Therefore, the objectives of this study were to (1) sequence and analyze the SFB genome from SFB isolated from Lohmann Select Leghorn (LSL) chickens; (2) compare the metabolism and phylogenetic relationships of SFB sequenced from chickens (LSL-SFB) to those of other avian species (turkeys) and mammalian hosts (humans and rodents); and (3) detect and compare key immune effector genes of SFB in different chicken genetic lines [25,26].

## 2. Materials and Methods

### 2.1. Ethics Statement

Animal experiments were conducted following the guidelines set forth in the “American Veterinary Medical Association Guidelines, 2013.” To minimize stress during the experimental procedures, enrichment materials were added to the open floor pens. These animal experiments were approved by the Iowa State University Institutional Animal Care and Use Committee, under Log Numbers 18-386 (approved on 20 June 2019) and 19-072 (approved on 21 June 2021).

### 2.2. Primary Collection of SFB from Conventional Layer Hen and Broiler Chickens

Fourteen-day-old Dekalb White Leghorn hens, Lohmann Select Leghorn hens, and Cobb700 broilers were transferred from Iowa commercial facilities to Iowa State University and euthanized via CO_2_ asphyxiation. Day-old Ross308 broilers were maintained at Iowa State University as previously described [6] and euthanized as above at 4 weeks post-hatch. All subsequent dissection took place in a biosafety cabinet.

### 2.3. Confirmation of SFB in Ilea Scrapings via PCR

DNA was extracted from distal ileum scrapings from 20 birds per genetic line to determine the distribution of SFB via boiling lysis [6]. PCR detection of SFB prior to sequencing was performed utilizing GoTaq Green Master Mix (Thermo Scientific, Waltham, MA, USA) utilizing SFB-specific primers as previously described [6].

### 2.4. Fluorescent In Situ Hybridization (FISH) and Gram-Stain Identification of SFB

For Gram-stain confirmation of SFB in ilea scrapings, 20 μL of stored scrapings were spotted onto glass slides. FISH was performed based on methods described previously [6,27] with minor modifications. After FISH, samples were counterstained with DAPI (4′,6-diamidino-2-phenylindole) at a final concentration of 5 mg/mL for 30 min at room temperature in the dark. Solutions were pelleted at 10,000× *g* and rinsed with phosphate-buffered saline (PBS) two times. Gram-stain and fluorescence microscopy were performed utilizing a Keyence BZ-X800 microscope (Keyence, Osaka, Japan). SFB-specific fluorescent microscopy images were overlaid with DAPI counterstained microscopy to demonstrate the total cell population in the images. All images were collected at 100× magnification.

### 2.5. Nanopore and Illumina Sequencing

DNA from ileal scraping was extracted using the PacBio Nanobind CBB Kit (Menlo Park, CA, USA) (kit # 102-301-900) and following the kit instructions except for the following: the samples were pretreated with Sigma St. Louis, MO, USA, Metapolyzyme enzyme mixture (product # MAC4L-5MG) instead of lysozyme. Metapolyzyme was dissolved in PBS at a concentration of 5 mg/mL and stored at −80 °C until needed. Resuspended samples were mixed with 20 μL of Metapolyzyme by pulsed vortexing 10 times and pipetting, followed by brief centrifugation. The samples were incubated at 35 °C for 15 min. The Nanobind CBB protocol for “Extracting HMW DNA from Gram-positive bacteria using PacBio, Menlo Park, CA, USA, Nanobind kits” was followed (protocol # 102-573-900).

High molecular weight DNA was quantified by Qubit using an Invitrogen (Thermo Fisher Scientific), Waltham, MA, USA, Qubit dsDNA broad range assay kit (Q32853), and fragment length was determined using an Agilent Technologies, Santa Clara, CA, USA, 5200 Fragment Analyzer with kit DNF 464-0500. To help eliminate low molecular weight DNA from the samples, a PacBio, Menlo Park, CA, USA, Short Read Eliminator kit (102-208-300) was used according to the manufacturer’s protocol. Qubit and Fragment Analyzer were performed again to determine the DNA concentration and fragment length. All processes were performed according to the kit instructions.

Libraries for Oxford Nanopore sequencing were prepared using the Oxford Nanopore, Oxford, UK, Native Library Kit (SQK-LSK109) and the Oxford Nanopore, Oxford, UK, Native Barcoding Expansion 1–12 kit (EXP-NDB104), following the kit instructions. Hi-C library production was performed using the Phase Genomics, Seattle, WA, USA, Proximo Hi-C Microbe Kit (KT1045) following the manufacturer’s protocol.

Oxford Nanopore sequencing was performed on a GridION sequencer using an Oxford Nanopore, Oxford, UK, R9.4.1 Flow Cell (FLO-MIN106D) and following kit instructions. Illumina Inc., San Diego, CA, USA, NovaSeq 6000 sequencing was performed for the Hi-C libraries using 150-cycle paired-end sequencing on a single lane of an SP flow cell (20028400), following the kit’s instructions.

### 2.6. Assembly of LSL-SFB Genome

The initial step involved aligning chicken gut metagenomic HiC reads to the reference chicken genome (*Gallus gallus*, GRCg7b) using the Burrows–Wheeler Aligner (BWA) version 0.7.17 [28]. This alignment facilitated the filtering of host reads, allowing for a more targeted assembly of the SFB genome. Subsequently, the aligned reads were processed with Samtools (v1.19.1) to generate a sorted BAM file, which was then indexed to facilitate efficient access and manipulation. To specifically isolate the SFB genomic sequences from turkey [18], which were hypothesized to be closely related to those in chicken, we extracted reads aligning to known turkey SFB contigs from the sorted BAM file. This extraction yielded a BAM file containing only turkey SFB sequences, which were then indexed for downstream analyses. To reconstruct the LSL-SFB genome, we employed the “samtools consensus” command [29], which generated a consensus sequence representing the SFB genome assembly. This consensus sequence served as a scaffold for further refinement.

### 2.7. HiC-Based Scaffolding

The assembly process was enhanced by incorporating Hi-C data to scaffold the genome to a higher contiguity. The Hi-C reads were aligned to the initial SFB consensus scaffolds using BWA. The alignment data were processed with Juicer (version 1.5.7) to identify chromatin interactions within the LSL-SFB genome. The 3D-DNA pipeline (version 180114) was then used to create visualizations of scaffolding based on the Hi-C interaction data. Finally, JuiceBox (version 1.11) [30] provided a graphical interface to manually refine the assembly using a 3D genome structure. Initial scaffolding with 3D-DNA presented a confident arrangement of these 41 contigs, ensuring an accurate representation of the LSL-SFB genome. The integration of Hi-C data enabled the construction of a comprehensive genome assembly of the SFB present in the chicken gut, offering insights into its genomic structure and potential functional capabilities within the host’s microbiome. Genome completeness was estimated using CheckM v1.2.2 and Anvi’o v7.1, which determine completeness based on lineage-specific single-copy marker genes following MIMAG standards [31].

The finalized genome sequence of LSL-SFB, obtained through HiC assembly, was annotated using Prokka (version 1.14.6) under default parameters. The annotation workflow in Prokka encompasses several steps: tRNA genes were detected with Aragorn (version 1.2.41), rRNA genes were predicted using Barrnap (version 0.9, available at https://github.com/tseemann/barrnap accessed on 22 August 2023), open reading frames were identified with prodigal (version 2.6.3), protein homology was assessed through BLASTp (version 2.14.0), and domain-based annotation was conducted using HMMER (version 3.3.2). Subsequent annotations of the protein sequences identified by Prokka were conducted with eggnog-mapper (version 2.1.12) with default parameters against the eggNOG database (version 5).

### 2.8. Comparative Pangenomic Analysis

Comparative pangenomic analysis was performed using Anvi’o v8 [32] on seven SFB genomes: chicken SFB (this study), turkey SFB (GCA_001655775.1), mouse SFB-Yit (AP012209.1), mouse SFB-Japan (AP012210.1), mouse SFB-NL (NZ_CP008713.1), rat SFB-Yit (AP012211.1), and human SFB (GCA_902374285.1). All genome FASTA files were reformatted to comply with Anvi’o’s simple defline requirements using ‘anvi-script-reformat-fasta’ with the ‘--simplify-names’ flag, and contig databases were generated for each genome using ‘anvi-gen-contigs-database’. Each contig database was functionally annotated with Hidden Markov Models (HMMs) for bacterial single-copy core genes (‘anvi-run-hmms’), COG20 functional categories (‘anvi-run-ncbi-cogs’), tRNA genes (‘anvi-scan-trnas’), and single-copy gene (SCG) taxonomy (‘anvi-run-scg-taxonomy’), all with 4 threads. A genome storage database containing all seven SFB genomes was created using ‘anvi-gen-genomes-storage’, and the pangenome was computed using ‘anvi-pan-genome’ with default parameters (MCL inflation: 2.0, minbit heuristic: 0.5) and 10 threads. Gene clusters were identified based on amino acid sequence similarity using DIAMOND BLASTP (bundled with the anvi’o software v7.1) and organized using the Markov Cluster Algorithm (MCL). The average nucleotide identity (ANI) between genomes was calculated using ‘anvi-compute-genome-similarity’ with the pyANI algorithm and integrated into the pangenome database. The pangenome was visualized using ‘anvi-display-pan’.

### 2.9. Metabolic Analysis of LSL-SFB Genome and Comparisons to SFB from Other Host Species

The complete genome of the LSL-SFB was analyzed for the presence and completion of metabolic networks via Pathway Tools software (version 27.1; [32]). The metabolic networks were compared to publicly available and previously annotated SFB genomes (mice and rats) and control organisms such as *Escherichia coli* K-12 MG1655, *Clostridium perfringens* ATCC13124, and *Bacillus cereus* ATCC10876 that represent well understood metabolic models, organisms that are somewhat related to SFB, and organisms that are often found in the same gastrointestinal niche as SFB, respectively, that are all readily accessible in the MetaCyc database [24]. The number of enzymes recognized in biosynthetic, degradation/utilization, and transport were enumerated and normalized to those found in *E. coli* K-12 MG1655.

### 2.10. Amplification and Cloning of LSL-SFB fliC

A flagellin subunit (*fliC-2*) was uniquely identified from the LSL-SFB annotated genome. DNA isolated from four birds per genetic line (White Dekalb, LSL, Ross308, and Cobb700) were screened for the presence of this *fliC-2* via PCR using primer pairs generated to amplify the entire gene: F: 5′ ATGGATAGTTTTACTTTTAATACTAATGTTGGTGGTAATG-3′ and R: 5′ TCTTAAGATAGATAAAACTTGTTGTGGTGCTTGGTTATAA-3′ and Phusion High-Fidelity Master Mix (Thermo Scientific, Waltham, MA, USA). Following detection, the gene was amplified using Phusion High Fidelity polymerase (Thermo Scientific, Waltham, MA, USA) and primers designed for blunt end cloning into the pET101-d/TOPO vector (Thermo Scientific, Waltham, MA, USA). After cloning, probable transformants were screened via PCR, amplifying the T7 region using Phusion High Fidelity polymerase (Thermo Scientific, Waltham, MA, USA) per kit protocol. The resulting PCR amplicon was submitted for Sanger sequencing at the Iowa State DNA Facility from three positive clones per genetic line. Consensus sequences of each *fliC-2* from each genetic line were created using the triplicate results in Benchling (benchling.com) using the MAFFT algorithm. Consensus sequences were then aligned to the predicted *fliC-2* generated from the metagenomic sequencing utilizing the Molecular Evolutionary Analysis (MEGA, Version 11) [33] to identify single nucleotide polymorphisms (SNPs), changes in amino acid residues, and homology amongst chicken SFB from different genetic lines.

### 2.11. Phylogenetic Relationship of Chicken SFB fliC-2

Using the Basic Local Alignment Search Tool (BLAST) (BLAST+ 2.14.1), we queried the predicted *fliC-2* sequence derived from metagenome sequencing against both the BLASTn (nucleotide) (version 2.16.0+) and BLASTp (protein) (version 2.10) databases. This process allows us to identify the top 20 aligned sequences related to both SFB and non-SFB sequences. The tree with the highest log likelihood (−3354.69) is shown. The percentage of trees in which the associated taxa clustered together is shown next to the branches. Initial tree(s) for the heuristic search were obtained automatically by applying Neighbor-Join and BioNJ algorithms to a matrix of pairwise distances estimated using the Tamura–Nei model [34] and then selecting the topology with the superior log likelihood value. The evolutionary history was inferred using the Maximum Likelihood method and JTT matrix-based model [35]. Initial tree(s) for the heuristic search were obtained automatically by applying Neighbor-Join and BioNJ algorithms to a matrix of pairwise distances estimated using the JTT model and then selecting the topology with the superior log likelihood value. All analyses were performed in MEGA 11 software [33].

### 2.12. Generation of Chicken SFB Three-Dimensional Models and Interactions with Chicken TLR-5

To analyze the impact of SNPs and amino acid substitutions on the protein structures of the chicken SFB FliC-2 across all four genetic lines, we used AlphaFold2 for protein structure prediction. AlphaFold2 generates five structural models for each input sequence and ranks them based on an internal confidence metric, primarily the predicted Local Distance Difference Test (pLDDT) score. We selected the model with the highest ranking as the final structure for analysis, following AlphaFold2 default recommendations. The structures were visualized using UCSF ChimeraX (version 1.7, https://www.cgl.ucsf.edu/chimerax/ accessed on 30 August 2024 [36,37,38]. Additionally, we displayed the best predicted interaction of the FliC-2 protein from each genetic line with the chicken TLR-5-protein (UniProt ID C4PCK0; [39]).

### 2.13. Predicted Cross-Species Interaction of LSL-SFB, Mouse SFB, and Host TLR-5s

To understand the binding dynamics of SFB flagellin from different sources, the LSL-SFB FliC-1 and FliC-2 and homologous SFB FliC from mice were computationally bound to either the chicken or mouse TLR-5 (UniProt ID Q9JLF7; [40]). The sequences were submitted to the HDOCK integrated protein–protein docking server (http://hdock.phys.hust.edu.cn/ accessed on 30 August 2024) [41,42]. Visualization of resultant interactions and binding distances were generated for each protein–protein interaction using BioPython version 1.83 [42].

## 3. Results and Discussion

### 3.1. Detection and Collection of Avian-Specific SFB from Different Chicken Genetic Lines

**Figure 1:**
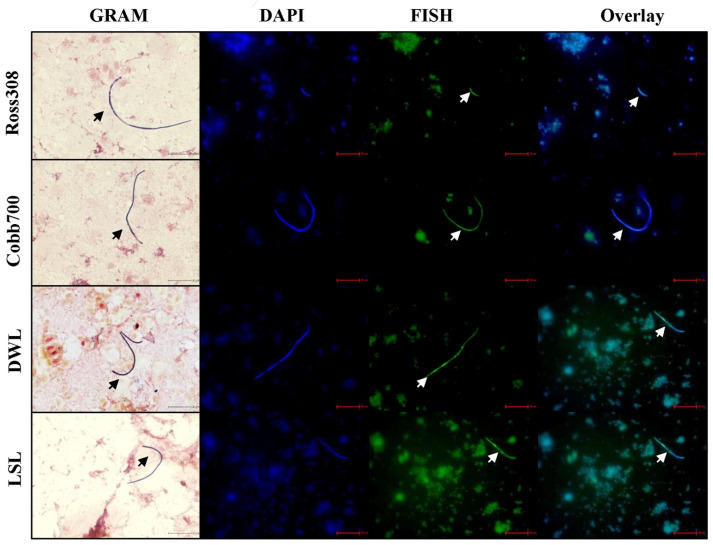
Microscopy detection of segmented filamentous bacteria in distinct chicken genetic lines. Distal ileum scrapings from Ross308, Cobb700, Dekalb White Leghorn (DWL), and Lohmann Selected Leghorn (LSL) chickens were subjected to Gram-stain, segmented filamentous bacteria (SFB) specific fluorescent in situ hybridization (FISH), and DAPI (4′,6-diamidino-2-phenylindole) microscopy. Images were collected at 100× magnification using the Keyence BZ-X800. Scale bars represent 20 μm. Arrows indicate the filamentous form of SFB.

Primary studies on SFB were done in rodents [1,21,43]. Recently, our team and others have detected the presence of SFB in avian species, including turkeys [18] and chicken layers [Dekalb White Leghorn (DW)] and broilers (Ross308, and Hubbard) [5,6,12,27], through PCR and microscopy. A recent study comparing SFB from mice, rats, humans, and turkeys showed lineages in SFB adapted to different hosts, and cross-colonization of SFB from mice to rats and vice versa resulted in SFB becoming undetectable in the alternative host, indicating SFB host-specificity [10,13,17]. However, it is unknown whether avian SFB from different genetic lines are identical. This study thus aimed to compare SFB from two layer (DW and LSL) and two broiler (Ross 308 and Cobb 700) genetic lines. Testing ileal scraping from different genetic lines detected the presence of SFB by PCR and the SFB filamentous morphologies were detected in both Gram-stain and FISH microscopy of the distal ileum (Figure 1), indicating the completion of the unique lifecycle of SFB in its specific host maturing from intracellular offspring or spores to filamentous SFB, which is occurring only when in contact with host epithelial. This provides useful identification of SFB obtained from a host that are specific to that host [6,11,27]. These SFB-enriched ileal scrapings were then used for SFB sequencing, as described below.

### 3.2. Generation and Assembly of the First Chicken SFB Genome

Genomic DNA was successfully isolated from SFB ileal samples collected from LSL chickens. Utilizing high-throughput sequencing technologies, a significant amount of raw sequence data was generated. Despite using both Nanopore and HiC sequencing techniques to increase the likelihood of a complete assembly, de-novo assembly attempts were unsuccessful. We performed preliminary de novo metagenomic assemblies using Flye (version 2.9.2, –meta mode) and Pomoxis to assess the feasibility of assembling the SFB genome without a reference. However, the high complexity of the chicken gut microbiome prevented complete recovery of the SFB genome. To evaluate SFB representation in the Nanopore data, reads were mapped to the turkey SFB reference genome using minimap2 (version 2.24), resulting in 210 primary alignments representing only 0.15% of total Nanopore reads. This low mapping fraction confirmed that SFB is a rare member of the chicken gut community and explained the failure of de novo assembly from Nanopore data alone. The mapped Nanopore reads covered 38 of 41 turkey SFB contigs with variable depth (1–46×), while three contigs showed no coverage. The Nanopore-based consensus spanned approximately 1.2 Mb compared with the expected 1.6 Mb genome size, indicating insufficient depth for complete reconstruction. These results demonstrated that the Nanopore data were valuable primarily for confirming SFB presence and estimating genomic coverage. Consequently, we relied on Hi-C short-read data, which provided ~18× coverage across the genome, for reference-guided consensus generation of the chicken SFB genome. Using these alignments, we created consensus sequences with Samtools, resulting in the initial reference-guided assembly of the LSL-SFB genome. This initial assembly, prior to Hi-C scaffolding, yielded 41 scaffolds, totaling 1,612,002 base pairs, with a mean scaffold size of 39,317 bp and a median size of 29,165 bp (Table 1)-. Following Hi-C-based scaffolding and manual curation through the Juicer and 3D-DNA pipelines, we consolidated the 41 scaffolds into a single pseudomolecule of 1,631,855 bp (Table 2). Table 2 compares the assembly metrics before and after Hi-C scaffolding, showcasing significant improvements in contiguity (41 contigs reduced to 1 contig), gene annotation completeness (1502–1529 coding sequences), and RNA gene identification (1–2 rRNA genes; 33–35 tRNA genes). The final scaffolded LSL-SFB genome size (1,631,855 bp) is highly comparable to the turkey SFB reference genome (1,631,326 bp) [18], supporting the biological validity of our assembly (Table 3).

**Table 1:** Assembly statistics for the chicken SFB genome using turkey SFB as a reference.

Metric	Value
Number of scaffolds	41
Total size (bp)	1,612,002
Longest scaffold (bp)	138,357
Shortest scaffold (bp)	905
Mean scaffold size (bp)	39,317
Median scaffold size (bp)	29,165
N50 value (bp)	68,453
GC content (%)	~25

**Table 2:** Comparison of the chicken SFB genome before and after Hi-C assembly.

Metric	Before Hi-C Assembly	After Hi-C Assembly
Contigs	41	1
Bases	1,612,002	1,631,855
CDS	1502	1529
rRNA	1	2
tRNA	33	35
tmRNA	1	1

**Table 3:** Annotation comparison between chicken and turkey SFB genomes.

Metric	LSL-SFB Genome	Turkey SFB Genome
Number of Contigs	1	41
Total size (bp)	1,631,855	1,631,326
Number of Coding sequences (CDS)	1529	1570
rRNA genes	2	1
tRNA genes	35	33
tmRNA genes	1	1

The scaffold composition was predominantly adenine and thymine, contributing to an average GC content of approximately 25%. The completeness of the turkey SFB genome assembly was assessed using the BUSCO bacteria_odb10 dataset, achieving a 90.3% completeness score with no duplicated BUSCOs. The LSL-SFB genome, however, scored slightly lower at 83.9% completeness, with a small portion of the genome (4.8%) identified as fragmented and 11.3% missing BUSCOs.

Genome completeness was estimated using CheckM v1.2.2 and the Anvi’o v7.1 ‘estimate_genome_completeness’ function, which yielded values of 90.22% and 91.55%, respectively. Both approaches rely on analyzing lineage-specific marker genes, and the close agreement between the results indicates a reliable assessment of completeness. BUSCO analyses using the bacteria_odb12 and firmicutes_odb10 datasets yielded lower completeness values of 86.2% and 83.9%, respectively. These discrepancies are likely due to differences in the marker gene sets and lineage representation within the OrthoDB database. Since CheckM and Anvi’o are specifically designed for prokaryotic genomes, their estimates were used as the primary measure of genome quality. This approach is consistent with the MIMAG (Minimum Information about a Metagenome-Assembled Genome) and MISAG standards published by the Genomic Standards Consortium, which define high-quality bacterial genomes as having ≥90% completeness and ≤5% contamination, as determined by tools such as CheckM [31].

This study presents the first gene annotation of avian SFB genomes from a chicken (this study) and turkey (GCA_001655775.1). The SFB turkey genome had slightly more coding sequences, with 1570, compared to 1529 in the LSL-SFB genome. Both genomes had consistent annotations of other genomic features, such as rRNA, tRNA, and tmRNA (Table 3).

Further quality assessments involved polishing the LSL-SFB genome using Polca (POLCA bundled internally with MaSuRCA v4.0.5), which led to minor modifications in the assembly. The total base count was reduced to 1,611,895, and the number of coding sequences increased from 1502 to 1529, while BUSCO scores indicated a slight decline in completeness from 83.9% to 83.1% and an increase in fragmented BUSCOs. This suggests that polishing may have over-corrected regions of the genome.

**Figure 2:**
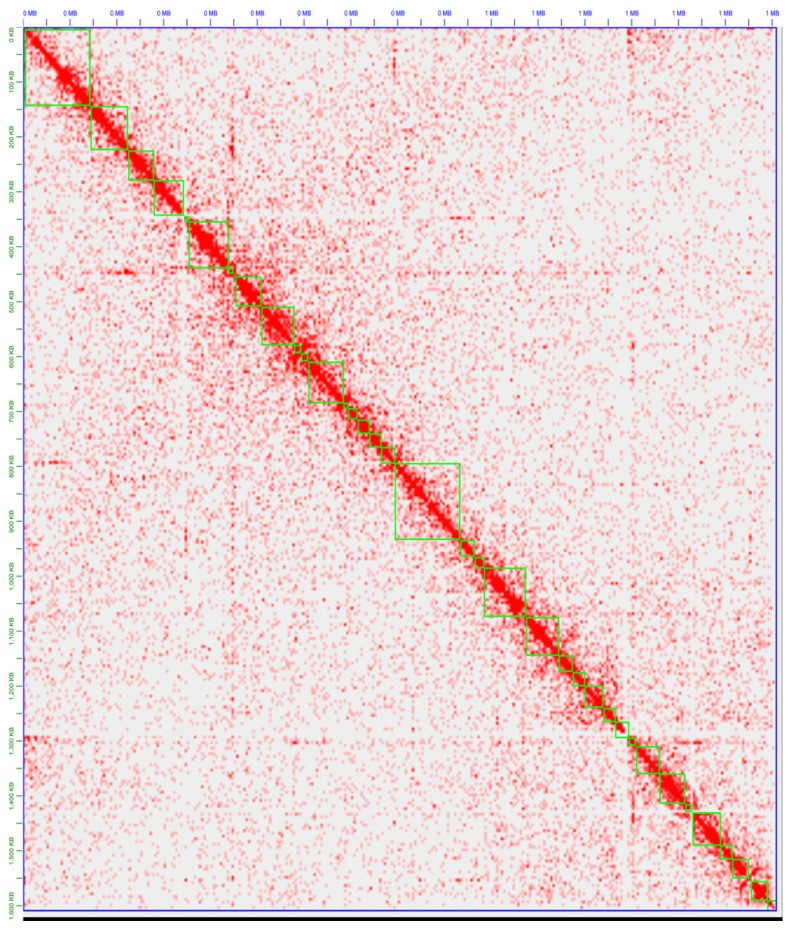
Juicebox Hi-C contact map of LSL-SFB at 5kb resolution without normalization. Red indicates Hi-C interactions between contigs, with green boxes indicating scaffolded contig connections. Notably, all contigs exhibit a Hi-C signal, with higher intensity signal between contigs that should be close in the genome in proximity. Using this justification, we split and rescaffolded multiple contigs to better represent the genomic DNA of LSL-SFB.

The polished LSL-SFB genome assembly was then scaffolded with Hi-C using the Juicer/3d-DNA/Juicebox pipeline, consolidating 41 contigs into one pseudomolecule. Hi-C is a chromosome conformation capture technique that preserves the spatial proximity information between DNA sequences. In our metagenomic study, Hi-C served multiple critical functions: (1) Metagenomic binning: Hi-C proximity ligation preferentially links DNA fragments that are physically close to each other within the same cell. This means that DNA from the same bacterial species shows more frequent Hi-C linkages than DNA from different species. We used this property to identify and bin SFB-specific reads from the complex chicken gut metagenome; (2) Scaffolding information: Hi-C provided long-range connectivity data, which allowed us to order and orient contigs. As a result, we successfully consolidated 41 initial contigs into a single pseudomolecule (Table 2). The Hi-C contact map (Figure 2) visually illustrates the chromatin architecture and validates the accuracy of our scaffolding; (3) Coverage assessment: Aligning Hi-C reads to the turkey SFB reference genome revealed excellent coverage (an average of ~18×), across all contigs. This demonstrates that we successfully captured the SFB genomic material, despite its low abundance within the overall community; and (4) Validation of genome organization: The strong diagonal signal in our Hi-C contact map (Figure 2), along with the off-diagonal interaction patterns, confirms that our assembled genome represents a single, circularized chromosome consistent with what is known about SFB biology.

The Hi-C contact map shown represents the 3D architecture of the LSL-SFB genome (Figure 2). The intensities of red along the diagonal indicate regions of high interaction frequency and closeness in proximity, thus displaying the genome’s high contiguity. Multiple reference-based contigs required rearrangement to enable this contiguity, likely representing a biological difference in genome organization between turkey and chicken SFB genomes. The off-diagonal spots of higher intensity suggest long-range interactions, which are significant for understanding the functional organization within the circular genome. These interactions might represent regulatory regions or structural elements essential for maintaining the stability and functionality of the SFB genome. This assembly process utilizes Hi-C data from metagenomic samples, providing a strong method for elucidating the genome architecture of complex microbial entities, such as the SFB within a mixed microbial community. This refined SFB genome assembly aligns with our objective to develop robust, replicable methods that will serve as a benchmark for future SFB genomic studies.

### 3.3. Pangenomic Analysis of SFB

During the review of this manuscript, a study was published reporting the sequencing of the genomes of two chicken SFB included in their treatments [44]. However, these SFB were not confirmed to be specific to chickens, as in vivo colonization, a process that leads to the formation of segmented filaments in their specific host only, was not conducted [5,6]. The detection in chickens of two different SFBs in that study raises concerns about potential contamination from other hosts. Additionally, it is important to note that these genomes have not yet been analyzed or made publicly accessible, so they were not included in our comparisons.

**Figure 3:**
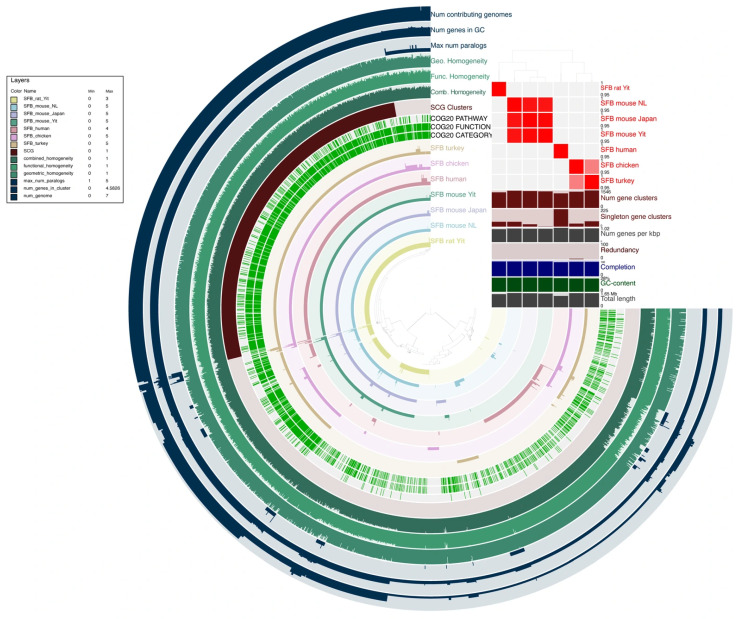
Comparative pangenomic analysis of segmented filamentous bacteria strains from mammalian and avian hosts. A pangenome graph was generated using Anvi’o with gene clusters arranged according to their genomic position in the reference genome of LSL-SFB (chicken). The concentric rings, starting from the center, represent gene presence or absence across the genomes of SFB-rat-Yit, SFB-mouse-NYU, SFB-mouse-Japan, SFB-mouse-Yit, SFB-human, SFB-turkey, and LSL-SFB. Additional layers include annotations for single-copy gene (SCG) clusters, functional classification based on the COG20 database (Pathway, Function, and Category), gene cluster size, the number of genomes contributing to each cluster, the maximum number of paralogs per cluster, and measures of gene cluster conservation including geometric homogeneity, functional homogeneity, and a combined homogeneity score. A hierarchical clustering dendrogram and heatmap (top right) display gene content similarity among the seven SFB genomes, with red intensity indicating higher similarity based on the Jaccard index. This clustering highlights a clear separation between avian SFB strains (LSL-SFB and SFB-turkey) and mammalian strains (mouse, rat, and human), suggesting host-specific genomic adaptation. Bar plots adjacent to the heatmap summarize genome-level statistics for each strain, including total number of gene clusters, singleton clusters, gene density (genes per kilobase), redundancy of SCGs, genome completeness, GC content, and total genome length.

In our study to investigate the genomic diversity and host-specific adaptations of SFB, we performed a comparative pangenomic analysis of seven SFB strains of mouse, rat, human, turkey, and chicken hosts, using LSL-SFB (chicken) as the reference genome for gene cluster ordering (Figure 3). The concentric rings in the pangenome visualization represent the presence or absence of gene clusters across the genomes, revealing both conserved and lineage-specific features. Overall, among the mammalian SFB strains, there was substantial conservation of core gene clusters, while LSL-SFB and SFB-turkey shared a distinct gene repertoire that diverged markedly from mammalian lineages. Interestingly, the avian SFB strains clustered into a separate clade in the phylogenomic tree and heatmap, consistent with strong gene content similarity and overlapping sets of genome-specific clusters. This distinct clustering supports the notion of host-specific adaptation and divergence, aligning with prior findings that avian-associated SFB harbor unique genes associated with niche-specific metabolic functions, such as glycan metabolism, biotin biosynthesis, and bile acid recycling [18]. These capabilities likely enable colonization and survival in the unique physiology of the avian gut, highlighting the influence of host-specific selective pressures on genome evolution. The human SFB strain also exhibited considerable genetic divergence, sharing only 65–71% average amino acid identity with other SFB genomes, a pattern that reinforces the presence of a human-adapted variant with unique genomic features [17]. Interestingly, despite overall conservation among rodent SFBs, strain-level polymorphisms and expansions of specific protein families were observed, particularly among the three mouse-derived strains, suggesting ongoing diversification even within a single host species [11].

Functional annotation of gene clusters using the COG20 database highlighted the potential functional divergence between SFB strains from different hosts (Figure 3). In particular, the presence of distinct COG20 annotations in gene clusters specific to avian strains (LSL-SFB and SFB-turkey) suggests functional specialization that may underlie differences in host–microbe interactions, including immunomodulatory effects, adhesion mechanisms, and metabolic capabilities. While our pangenome visualization reveals that many avian-specific clusters are annotated with unique COG terms, further enrichment analysis will be required to statistically validate whether these categories are significantly overrepresented in avian lineages. In contrast, mammalian SFBs shared well-documented core metabolic deficiencies, particularly in amino acid biosynthesis, reinforcing their dependence on host-derived nutrients [45]. These observations suggest that while core symbiotic functions are conserved, gene content variation reflects adaptive divergence to host-specific intestinal environments and immune pressures.

Further supporting this divergence, LSL-SFB contained a large number of unique gene clusters and exhibited several paralog expansions, as shown by elevated values in the outer rings corresponding to gene counts and paralog numbers. Although single-copy gene (SCG) clusters were broadly conserved across all genomes—suggesting overall completeness and high assembly quality—metrics of geometric, functional, and combined homogeneity revealed strain-specific variability, particularly in the avian lineages. Such variation indicates that while conserved genomic elements underpin shared symbiotic lifestyles, diversification in gene structure and function likely reflects adaptive evolution to different host ecologies. In addition to gene content, differences in structural and functional features, such as flagellin gene families, may also contribute to host-specific interactions. Previous studies have shown that flagellin diversity among SFB strains plays a role in adhesion and immune recognition [43], and our data support the idea that genomic divergence is mirrored at the protein-interaction level.

Together, these findings reveal that SFB strains have undergone substantial genomic diversification in response to host-specific environments. Avian SFB lineages exhibit the most pronounced divergence from their mammalian counterparts, both in gene content and functional classification. The combination of core gene conservation and accessory genome variability underscores a co-evolution model, whereby SFB have adapted their metabolic capabilities, adhesion properties, and immune evasion strategies to align with the selective pressures of their respective hosts.

### 3.4. Metabolic Analysis of LSL-SFB Compared to SFB from Other Hosts

A crucial limitation in our ability to study SFB is their uncultivability; as outside of mono-associated mice, SFB have reportedly only been cultured a single time in a limited capacity [1] that we could not successfully replicate with our avian SFB. To gain a clearer understanding of the metabolic needs of LSL-SFB, we utilized Pathway Tools to generate a metabolic network of LSL-SFB. Through metabolic network analysis, we identified 118 pathways, 814 enzymatic reactions, 69 transport reactions, 452 enzymes, and 101 transporters. As previously reported in mice SFB, LSL-SFB appear to have a heterotrophic lifestyle evidenced by nearly complete glycolysis and pentose phosphate pathways [11]. Similarly, the LSL-SFB genome did not display any genes encoding components of the electron transport chain. However, genes involved in fructose and mannose metabolism (fructokinase; mellata_01407), similar to those of murine SFB, were identified in LSL-SFB. But uniquely, the LSL-SFB genome contained genes responsible for the metabolism of lactose (b-galactosidase; mellata_00353) and sucrose (sucrose-6-phosphate hydrolase; mellata_01408). Genes encoding for bile acid degradation (3-dehydro bile acid delta (4,6)-reductase; mellata_01465) were also uniquely found in the LSL-SFB genome. The presence of enzymes capable of bile acid metabolism can elicit the ability of SFB to colonize the mucosa of the ileum [46,47,48].

Based on its genome, the biosynthetic capabilities of LSL-SFB are severely reduced. In the LSL-SFB genome, there were only genes encoding the biosynthesis of alanine, asparagine, aspartate, glutamate, isoleucine, lysine, and serine. Furthermore, only three forms of fatty acid and lipid biosynthesis pathways were identified in the LSL-SFB, e.g., palmitoyl-CoA, cardiolipin, and stearoyl-CoA. In addition, the LSL-SFB genome contains genes encoding for the synthesis of most nucleosides and nucleotides besides uracil, similar to murine SFB. Taken together, these results demonstrate the unique nutritional requirements of SFB and highlight its reliance on host metabolism for survival [1,11,49,50].

The direct interaction of SFB with the host epithelial tissue is hypothesized to be a key driver of the immune system maturation provided by the host–SFB crosstalk [51,52] However, one factor that has yet to be explored is the production of short-chain fatty acids (SCFA) by SFB. SCFAs like acetate and butyrate play a crucial role in immune cell development, maintenance of intestinal barrier integrity, and microbiome modulation [53,54]. Specifically in chickens, modulations in SCFAs have been shown to inhibit the transfer of bacterial antimicrobial resistance plasmids, inhibit the growth of *Salmonella*, and promote body weight gain in broiler chickens [55,56,57]. Uniquely, the LSL-SFB genome presents genes for pyruvate fermentation to acetate via a pyruvate ferredoxin oxidoreductase (mellata_01349). Although not considered a SCFA, a pathway to lactate production from pyruvate through a reversible reaction utilizing d-lactate dehydrogenase (mellata_00025) is encoded by the LSL-SFB genome. Generally associated with Lactic acid bacteria like *Lactobacillus* spp., the production of lactate is essential in promoting a healthy gut environment [58,59,60]. Specifically, increased lactate production from lactic acid bacteria has been shown to improve nutrient absorption and gut barrier function [61,62,63].

**Figure 4:**
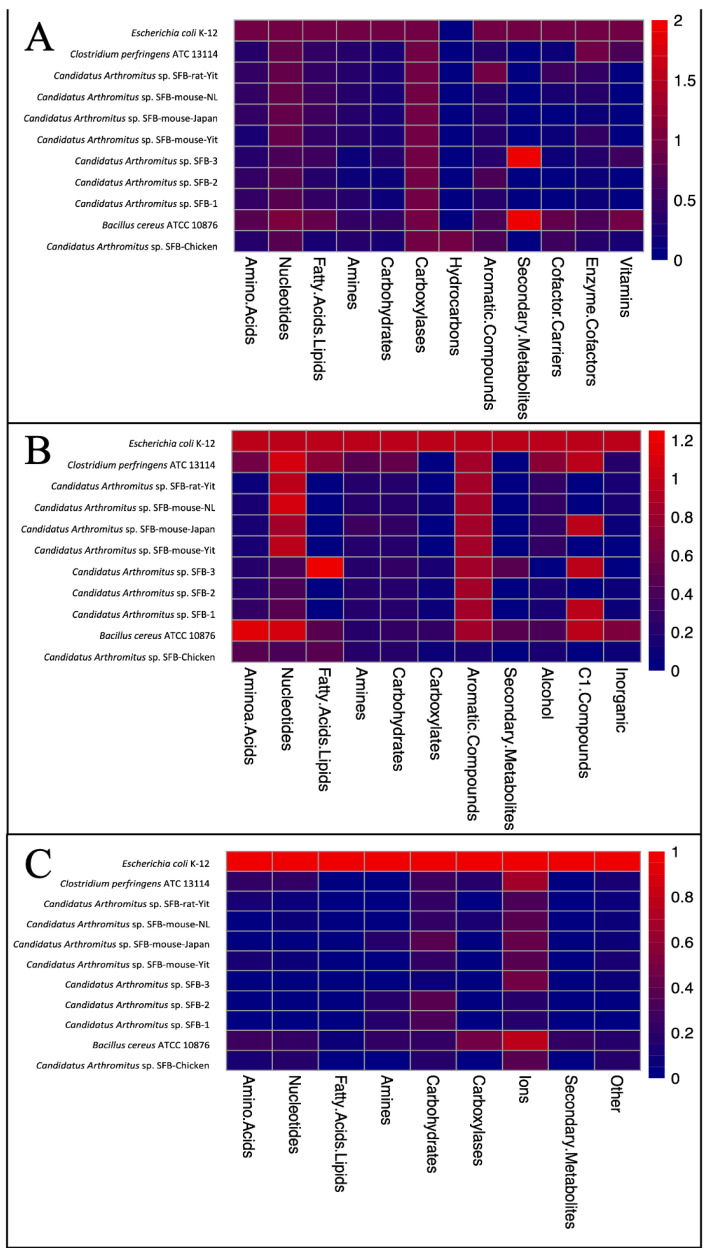
Metabolic network analysis of various segmented filamentous bacteria from different hosts. Various metabolic networks associated with (**A**) biosynthesis, (**B**) degradation and utilization, and (**C**) transport were enumerated via Pathway Tools from segmented filamentous bacteria (SFB) from mice, rats, and Lohmann Selected Leghorn chickens. The number of enzymes confirmed in given pathways were normalized to the control organism *E. coli* K-12 MG1655. Scale bars indicating color change represent the proportion of pathways in each compound to that of *E. coli*.

Since our attempts to culture LSL-SFB utilizing methods of murine in vitro culturing [1] have proven unsuccessful, we utilized Pathway Tools and MetaCyc to compare the metabolic networks of LSL-SFB and murine SFB. Total metabolic pathways were normalized to a control organism (*Escherichia coli* K-12 MG1655) to demonstrate the expected metabolism of a commensal, culturable organism. Furthermore, we included the metabolic networks of a bacterium distantly related to SFB, *Clostridium perfringens*, and *Bacillus cereus*, an organism often detected when collecting SFB from chicken intestines. In terms of biosynthetic pathways, LSL-SFB was comparable to those of murine SFB apart from LSL-SFB containing pathways involved in hydrocarbon biosynthesis and the lack of those involved in the production of secondary metabolites (Figure 4A). LSL-SFB demonstrated a higher propensity for amino acid degradation and utilization when compared to murine SFB but not aromatic compounds (Figure 4B). Finally, there were no observable trends in the presence of transporters across all SFB (Figure 4C). Further investigations into the unique metabolic needs of LSL-SFB are required to understand its difference from murine SFB and specific media requirements to promote its in vitro growth.

### 3.5. Structural and Phylogenetic Analysis of LSL-SFB FliC from Different Chicken Genetic Lines

Bacterial flagellin, a subunit of flagella, plays a crucial role in the interface of host–microbe interactions through Toll-like receptor 5 (TLR-5) [64]. Although this key receptor–ligand relationship is well-established, recent research has attempted to understand the fundamental functions of TLR-5 and its downstream signaling pathways passed the point of recognition [65]. Uniquely, the LSL SFB genome contains two flagellin subunit C (*fliC*) genes, including *fliC-1* (mellata_01007; ~1.2 kb), which is comparable in size to that of rodent SFB, and *fliC-2* (mellata_01003; 537 bp), which is smaller and absent in other sequenced SFB genomes. The nucleotide identity of aligned sequences of the LSL-SFB *fliC-1* and 2 and rodent-SFB fliC demonstrate that the *fliC* from rodents is significantly different than those of avian-SFB (Table 4).

**Figure 5:**
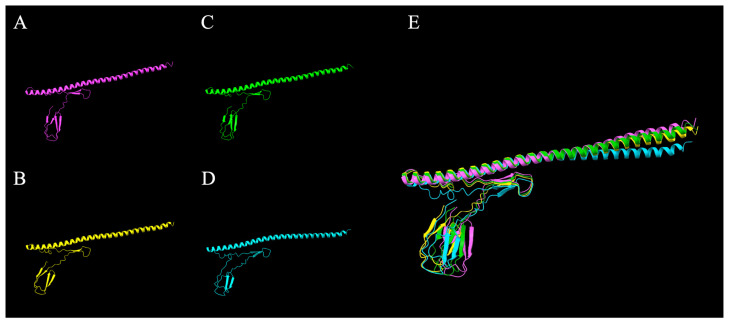
AlphaFold predictions of the FliC-2 protein structure. Sequences obtained from Sanger sequencing of the chicken SFB cloned fliC-2 from (**A**) Ross308, (**B**) Cobb700, (**C**) Lohmann Selected Leghorn, and (**D**) Dekalb White Leghorn SFB were utilized for AlphaFold protein structure prediction. All images were created using ChimerX software version 1.7. An overall of all protein structures (**E**) is provided.

**Figure 6:**
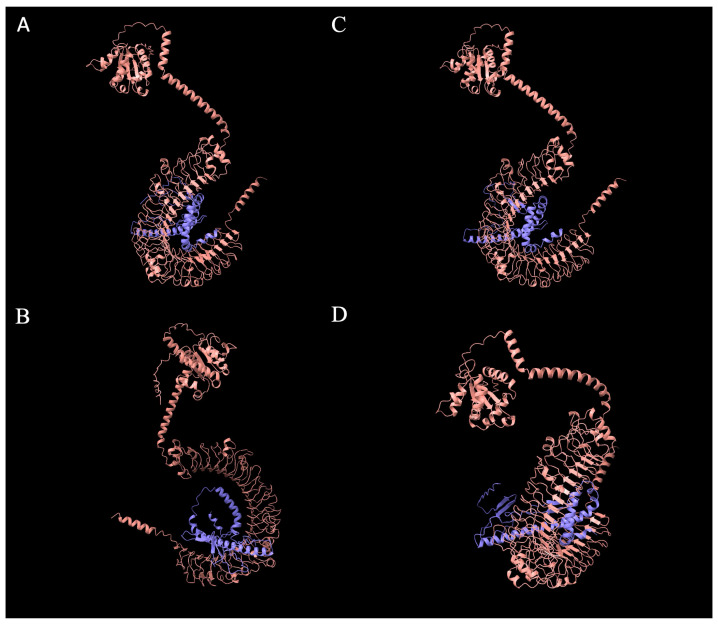
AlphaFold prediction of chicken SFB FliC-2 interaction with *Gallus gallus* TLR-5. *Gallus gallus* TLR-5 sequence (pink) was obtained from UniProt and used to predict the interaction with various chicken SFB FliC-2 proteins (purple) from (**A**) Ross308, (**B**) Cobb700, (**C**) Lohmann Selected Leghorn, and (**D**) Dekalb White Leghorn chickens using AlphaFold. All images generated in ChimeraX software version 1.7.

**Figure 7:**
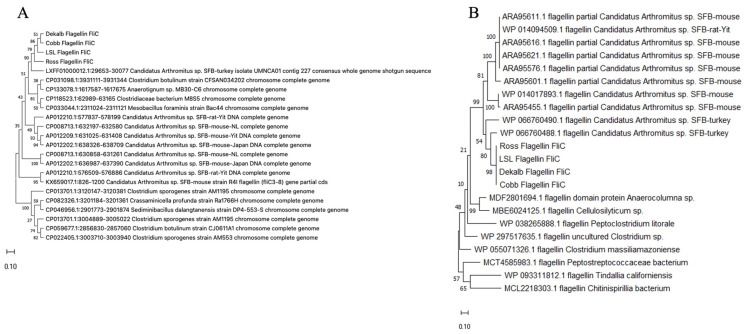
Phylogenetic analysis of flagellin subunit *fliC-2*. Phylogenetic comparisons of chicken SFB *fliC-2* nucleotide sequences (**A**) and FliC-2 amino acid sequences (**B**) to SFB sequenced from other sources and related *Clostridia* spp. (**A**) The tree with the highest log likelihood (−3354.69) is shown. The percentage of trees in which the associated taxa clustered together is shown next to the branches. This analysis involved 23 nucleotide sequences. There was a total of 726 positions in the final dataset. (**B**) The tree with the highest log likelihood (−7952.61) is shown. The percentage of trees in which the associated taxa clustered together is shown next to the branches. The tree is drawn to scale, with branch lengths measured in the number of substitutions per site. This analysis involved 22 amino acid sequences. There were a total of 649 positions in the final dataset. Evolutionary analyses were conducted in MEGA11.

Owing to the unique size of *fliC-2* and its analog absent in murine SFB, we investigated its presence and phylogeny amongst different chicken genetic lines of layers (LSL, DW) and broilers (Ross 308 and Cobb 700). As the whole gene of *fliC-2* was detected via PCR in all four chicken genetic lines tested, we next aimed to investigate whether the SFB from these different genetic lines demonstrated strain variation in the fliC-2 gene and constructed phylogenetic trees comparing both SFB and non-SFB species. The majority of the *fliC-2* sequence was conserved in all four genetic lines with a limited amount of single nucleotide polymorphisms (SNPs, Table 5). Next, we used AlphaFold to predict the folding and structure of the FliC-2 protein to distinguish if these SNPs impacted the overall structure of the FliC-2 protein from all four genetic lines or its interaction with chicken TLR-5. The SNPs made minor changes in protein folding in three-dimensional space (Figure 5A–E) but did not significantly impact the ability to interact with TLR-5 (Figure 6A–D). Through phylogenetic analysis of both nucleotide and predicted protein sequences, we demonstrated that all four chicken SFB *fliC-2* are clustered closely to sequences obtained from turkey SFB. The avian SFB show close relation to their murine counterparts compared to non-SFB *Clostridia* spp., further highlighting how SFB may have diverged in relation to host species’ evolutionary paths (Figure 7).

In the gastrointestinal tract, TLR-5 is expressed predominantly on the basolateral side of the intestinal epithelia compared to the epithelial cell surface in the respiratory tract [65,66,67]. This cellular localization is imperative for the ability of TLR-5 to recognize bacteria that have translocated across the epithelium to the basolateral tissue and distinguish between commensal gut microbiota and invading pathogens [68]. However, how commensal bacteria like SFB that produce flagellin avoid triggering inflammation is not fully understood. A recent study using recombinantly expressed mouse-SFB flagellins triggered comparable immune activation to SFB colonization in vivo. Furthermore, the data highlight how the introduction of mouse-SFB flagellin to mice induced the appearance of CD4+ T helper cells that produce IL-17 (TH17 cells) in the small intestinal lamina propria [25]. These findings indicate a highly structured relationship between SFB flagellin and the TLR-5 of their host. Recent data demonstrate that certain gut microbiota produce “silent” flagellin that are weak agonists of TLR-5 despite pattern recognition [69]. In addition, the interaction between TLR5 and flagellin, occurring at a site distinct from the pattern recognition domain, modulated receptor activity. This interaction was present in *Salmonella* flagellin but lacking in silent flagellins produced by commensal microbiota [69].

**Figure 8:**
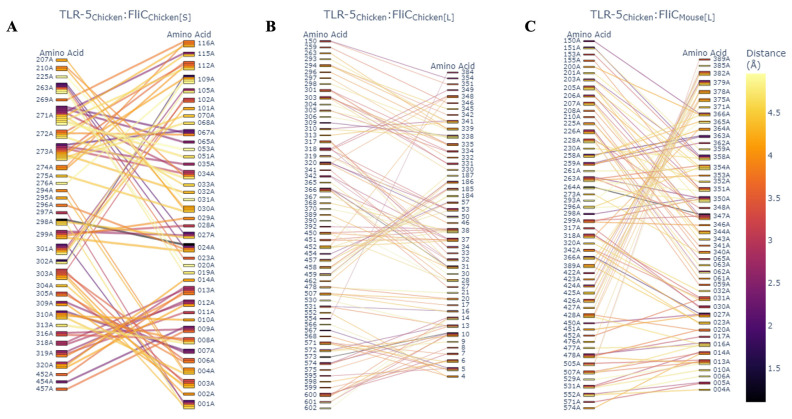
Parallel category plots of HDock interface residues. Interfaces between chicken TLR-5 binding and LSL-SFB FliC-2 (**A**), LSL-SFB FliC-1 (**B**) and mouse FliC (**C**). Color represents the computed interface distance between amino acids.

**Figure 9:**
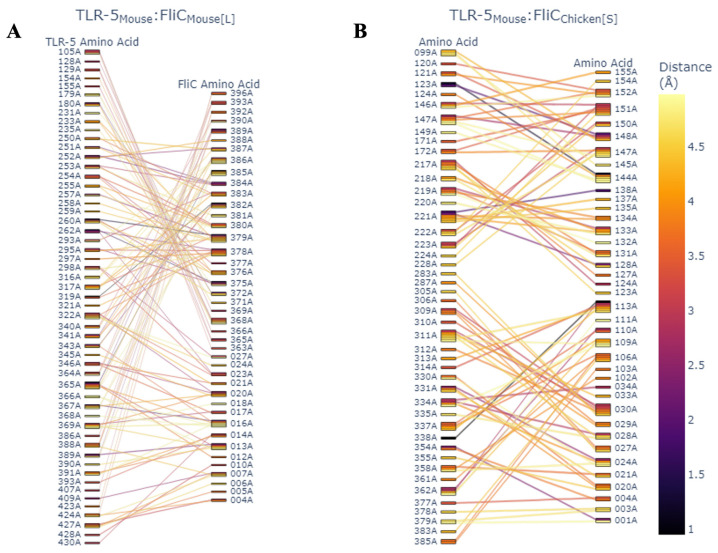
Parallel category plots of HDock interface residues. Interfaces between mouse TLR-5 binding and mouse SFB large FliC (**A**), and LSL-SFB FliC-2 (**B**). Color represents the computed interface distance between amino acids.

Finally, to investigate the role of SFB flagellin in host specificity, we utilized protein–protein docking via HDOCK [41] to give a detailed analysis of the residue–residue interaction of the LSL-SFB FliC-1 and FliC-2 and its mouse SFB counterpart with both chicken and mouse TLR-5. LSL-SFB FliC-2 demonstrated a much more interactive network of binding sites to chicken TLR-5 than LSL-SFB FliC-1 and murine SFB FliC counterparts (Figure 8A–C). Specifically, the number of interacting residues was increased in FliC-2, and the predicted distance of interaction was decreased, suggesting a closer binding interaction of chicken TLR-5. In the context of residue-specific interactions, it has been demonstrated that TLR-5 recognition of bacterial flagellin is dependent on leucine-rich repeats that are present in regions of residue 266–272, 383–387, and 442–446 of TLR-5 [64,70]. In this study, the LSL-SFB FliC-2 had the highest recognized binding to residues in these regions with the smallest distance of interaction where these interactions were absent in the mouse SFB binding to chicken TLR-5 (Figure 8A,B). Furthermore, when comparing the binding of the LSL-SFB FliC-2 to the mouse TLR-5, although the interaction distance was still closer than the mouse SFB, the interacting residues did not follow the same pattern observed when compared to chicken TLR-5 (Figure 9A,B). The authors postulate that this specific interaction between different types of flagellin could be key drivers for the host-specific immune maturation observed when SFB are able to colonize their respective hosts. Ongoing work utilizing protein purification is being utilized to better understand these interactions and elucidate mechanistic pathways for immune maturation in vitro.

## 4. Conclusions

Overall, our study provides new insights into the chicken SFB. The genome sequencing and analysis of the chicken SFB highlighted the divergence of the poultry SFB from that of the mammalians. The metabolic network analysis of the chicken SFB demonstrated reduced biosynthetic capabilities compared to that of mice SFB, indicating that SFB from different hosts exhibit different nutritional needs; thus, studies from one host may not be extrapolated to another host. This study identified a unique flagellin subunit in chicken SFB, in addition to *fliC-1* similar to that of mice, a significantly smaller flagellin subunit (*fliC-2*) was detected in chicken SFB only but not in previously sequenced SFB from other hosts, including turkey. The presence of the *fliC-2* gene was confirmed in different genetic lines of chickens tested, including layers (Lohmann Select Layer and Dekalb White) and broilers (Ross308 and Cobb700). Uniquely, our data show that SFB from different hosts differ significantly in relation to the genetic repertoire, metabolism, and effector proteins that are indicative of colonization and immunomodulation of the host. Understanding the routes of immunomodulation caused by SFB is imperative for improving gut immunity in chickens for animal health and consumer safety.

## Figures and Tables

**Table 4:** Nucleotide identity comparisons of various flagellin subunit (*fliC*) from chicken and rodent Segmented Filamentous Bacteria.

	LSL-*fliC*-*1*	LSL-*fliC*-*2*	Mouse-*fliC*	Rat-*fliC*
LSL-*fliC-1*		75%	82.09%	81.84%
LSL-*fliC-2*	75%		81.75%	81.60%
Mouse-*fliC*	82.09%	81.75%		99.93%
Rat-*fliC*	81.84%	81.60%	99.83%	
Accession Number	Mellata_01007	Mellata_01003	KX659017.1	AP012210.1

LSL, Lohmann Selcted Leghorn; two *fliC* (1 and 2) were detected in chicken SFB tested in this study.

**Table 5:** Nucleotide and amino acid identity of CSFB *fliC* detected in four chicken genetic lines.

Genetic Line	Nucleotide Identity (%)	Amino Acid Identity (%)
Lohmann Selected Leghorn	537/537 (100)	179/179 (100)
Dekalb White Leghorn	533/537 (99)	177/179 (99)
Cobb 700	530/537 (99)	175/179 (98)
Ross 308	520/537 (97)	177/179 (99)

## Data Availability

The original contributions presented in this study are included in the article. Further inquiries can be directed to the corresponding author.

## Abbreviations

The following abbreviations are used in this manuscript:

SFB	Segmented filamentous bacteria
LSL	Lohmann Select Layer
TLR	Toll-like Receptor
FliC	Flagellin subunit C
